# Prostate MRI for Improving Personalized Risk Prediction of Incontinence and Surgical Planning: The Role of Membranous Urethral Length Measurements and the Use of 3D Models

**DOI:** 10.3390/life13030830

**Published:** 2023-03-19

**Authors:** Thierry N. Boellaard, Marinus J. Hagens, Hans Veerman, Derya Yakar, Laura S. Mertens, Stijn W. T. P. J. Heijmink, Henk G. van der Poel, Pim J. van Leeuwen, Ivo G. Schoots, Margriet C. van Dijk-de Haan

**Affiliations:** 1 Department of Radiology, Netherlands Cancer Institute, Plesmanlaan 121, 1066 CX Amsterdam, The Netherlands; 2 Department of Urology, Netherlands Cancer Institute, Plesmanlaan 121, 1066 CX Amsterdam, The Netherlands; 3 Prostate Cancer Network the Netherlands, 1066 CX Amsterdam, The Netherlands; 4 Department of Urology, Amsterdam University Medical Centers, De Boelelaan 1117, 1081 HV Amsterdam, The Netherlands; 5 Medical Imaging Center, Departments of Radiology, Nuclear Medicine and Molecular Imaging, University Medical Center Groningen, University of Groningen, Hanzeplein 1, 9700 RB Groningen, The Netherlands; 6 Department of Radiology and Nuclear Medicine, Erasmus University Medical Center, 3015 GD Rotterdam, The Netherlands

**Keywords:** prostatic neoplasms, magnetic resonance imaging, prostatectomy, urinary continence, urethra, three-dimensional images

## Abstract

Prostate MRI has an important role in prostate cancer diagnosis and treatment, including detection, the targeting of prostate biopsies, staging and guiding radiotherapy and active surveillance. However, there are other ‘’less well-known’’ applications which are being studied and frequently used in our highly specialized medical center. In this review, we focus on two research topics that lie within the expertise of this study group: (1) anatomical parameters predicting the risk of urinary incontinence after radical prostatectomy, allowing more personalized shared decision-making, with special emphasis on the membranous urethral length (MUL); (2) the use of three-dimensional models to help the surgical planning. These models may be used for training, patient counselling, personalized estimation of nerve sparing and extracapsular extension and may help to achieve negative surgical margins and undetectable postoperative PSA values.

## 1. Introduction

Prostate magnetic resonance imaging (MRI) has an important role in the diagnosis and treatment of prostate cancer. The use of the Prostate Imaging Reporting and Data System (PI-RADS) assessment categories standardized the reporting for lesions within the prostate. The advent of MRI-directed targeted prostate biopsies improved the detection of clinically significant prostate cancer and lowered the detection of clinically insignificant cancer, compared with systematic biopsies [1,2]. Visual estimation of the tumor stage, mainly the T-stage, is known for its high specificity but low sensitivity for extracapsular extension [3]. Furthermore, prostate MRI is commonly used for guiding radiotherapy and active surveillance.

However, apart from these common applications, additional useful information can be extracted from the prostate MR images. One commonly studied aspect is the prediction of post-prostatectomy incontinence risk using measurements of (peri-)prostatic anatomical structures, which allows the better selection of patients suitable for radical prostatectomy [4]. Another interesting recent development is the use of 3D models for procedural planning and surgical guidance during radical prostatectomy [5,6]. Both topics are frequently used and analyzed at the highly specialized medical center to which this study group belongs.

Radical prostatectomy is increasingly being performed using a surgical robot (robot-assisted radical prostatectomy (RARP)). Radical prostatectomy aims to completely remove the prostate and prostate cancer with anatomical reconstruction of the vesico-urethral anastomotic complex. The goal is to achieve optimal oncological and functional outcome. Oncological outcome partly depends on the complete removal of the tumor, as positive surgical margins are correlated with biochemical recurrence [7]. The procedure may have a negative effect on urinary incontinence and erectile function, influencing the quality of life [8,9]. Illustrated by the fact that a substantial number of patients (3.43%) receive incontinence procedures after radical prostatectomy, such as a male sling [10].

Besides radical prostatectomy, other (treatment) options associated with lower rates of urinary incontinence can be considered, such as external beam radiotherapy, brachytherapy, and active surveillance [11]. Men with prostate cancer will be informed on the possible side effects of their therapeutic options before treatment. Based on this information, patients can decide which treatment is preferred (shared decision-making).

Preoperative planning with MRI may help to improve both oncologic and functional outcomes. However, the exact interpretation of MR images may be challenging for the urologist. It is a relatively technical analysis combining several MRI sequences (T1, T2, DWI and ADC, with or without DCE) that, in some cases, are challenging for even expert radiologists. Technical advantages have created the opportunity to create high-resolution MR images and 3D reconstructed models, either on the computer/tablet or using 3D-printing. To create these models, the prostate capsule, tumor and other structures are usually delineated by a radiologist. These segmentations are used to reconstruct 3D models with the use of (freely available) software. During RARP, the computer 3D models can even be viewed inside the console of the robot. The images can be viewed alongside the surgical field, or superimposed over the surgical field, the latter is called augmented reality (AR).

This narrative review will discuss the literature on: (1) the use of MRI-based (peri)prostatic anatomical measurements for improving personalized risk prediction of post-prostatectomy incontinence, most importantly, the membranous urethral length; (2) the use of MRI-based three-dimensional models to help procedural planning of radical prostatectomy.

## 2. Materials and Methods

We designed a narrative review focusing on the literature regarding anatomical factors on prostate MR related to post-prostatectomy incontinence and on the literature regarding the use of 3D models in surgical planning of a prostatectomy. A comprehensive search in the MEDLINE, Embase, Web of Science Core Collection, Cochrane and Google databases was performed for the anatomical factors. This search was used for a recent meta-analysis of our group [4] and was updated for this review on 5 August 2022. We added this search as Appendix A. The following PubMed search was used for articles on 3D models: (3D OR three dimensional OR Three-dimensional) AND (model OR printer OR printed) AND (prostatectom* OR RARP OR RALP), performed in December 2022. The references of the selected articles were checked.

## 3. Anatomical Parameters to Predict Urinary Continence after Radical Prostatectomy

Several patient-related factors are known to influence risk of urinary incontinence, such as age, pre-existing lower urinary tract symptoms and body mass index, and also, surgical factors have influence, for example, nerve sparing [12,13]. These factors can be considered for a personalized risk prediction on urinary in continence after prostatectomy. Next to clinical factors, prostate MRI parameters can improve personalized urinary incontinence risk prediction after radical prostatectomy. Because the MUL is by far the most studied parameter and also has a large influence on the predicted incontinence, we provide the most data on the MUL [4]. For other anatomical parameters, the evidence is still limited.

### 3.1. Membranous Urethral Length

**Figure 1:**
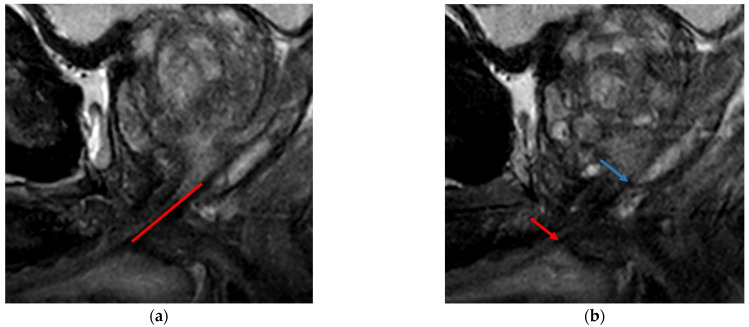
Membranous urethral length (MUL) measurement. (**a**) Midsagittal T2 weighted MRI image (**b**) Parasagittal T2 weighted MRI image. The measurement line of the MUL is shown with the red line. Regularly the lower border of the prostate (blue arrow) and upper border of the penile base (red arrow) can be better appreciated on the parasagittal images. In our center, we measure the MUL dorsal of (and parallel to) the hyperintense urethra lumen. Although this patient has a relatively long MUL, he used one pad a day after 12 months, with a score of 6 on 21 point scale for incontinence (0 = continent, 21 = very incontinent).

The membranous urethra is defined as the part of the urethra between the prostatic apex and the bulbus penis (bulb of the corpus spongiosum). The smooth muscle fibers of the inner (lisso)sphincter of the urethra and the striated muscle fibers of the outer (rhabdo)sphincter are important for preserving and regaining urinary continence after radical prostatectomy. The inner (lisso)sphincter extends from the vesical orifice to the perineal membrane whereas the outer (rhabdo)sphincter is the thickest at the level of the membranous urethra [14]. Given that, after radical prostatectomy, the only remaining inner sphincter fibers, but also the thickest outer sphincter fibers, are located in the membranous urethra, the importance of its length for urinary continence is easily understood. See Figure 1 for an example of a MUL measurement.

#### 3.1.1. Membranous Urethral Length Predictive Power

To date, about 50 studies studied the relationship between MUL and post-prostatectomy incontinence. Several meta-analyses confirmed the predictive power of this parameter [4,12,15]. A recent systematic review and meta-analysis showed the significant relation between the MUL and the extent of urinary incontinence at 1, 3, 6 and 12 months after radical prostatectomy [4]. Only one study in this review provided data on the 24-month incontinence risk with significant relation. Mungovan et al. showed in their meta-analysis that each additional millimetre of MUL was associated with a faster return to continence (hazard ratio 1.05) [15]. So, although the MUL seems strongly related to postoperative urinary incontinence, long-term results are scarce. A study from our research group showed that this personalized risk indeed influences the treatment that is chosen [9]. Especially patients with high risk for urinary incontinence after radical prostatectomy switched more frequently than patients with intermediate or low risk.

#### 3.1.2. Membranous Urethral Length Observer Agreement

Because of the predictive power of the MUL, it is already used in clinical practise. A high interobserver agreement is important. Several studies have studied the observer agreement, which can be measured by the intraclass correlations coefficient (ICC). The agreement results are variable [16], from fair agreement (ICC 0.34–0.38) [17,18] to high agreement (ICC 0.89) [19]. Training has been shown to improve the interobserver agreement [18].

The studies on MUL have shown great variability in the performance of measurements, in either coronal images, sagittal images or both. The descriptions in the literature are very limited and therefore not reproducible. The variability is also illustrated by the large variation in mean and median MUL measurements among studies. For example, in two studies performed in the same country, the difference between median MUL was 10 mm [20,21]. Our group performed an agreement study and found high intra- and inter-observer agreement using well-defined landmarks and standardized measurement technique [22]. Although the MUL is often measured by MRI, MUL can also be measured by using ultrasound [23,24].

### 3.2. Other Anatomical Parameters

The clinical impact of many other MRI-based anatomical parameters has been studied. These can be divided into prostate-related parameters, urethra-related parameters and musculoskeletal-related parameters.

Several parameters have shown a significant association in uni- and/or multivariate analysis with later return to continence after radical prostatectomy in one or few studies; these are summarized in Table 1 [4].

The shape of the apex, the IPP and levator ani muscle thickness were studied in more than one study. All the other parameters were reported in one study only. The details of these studies, such as type of analysis and number of patients, can be found in a recently published systematic review [4].

Several of these correlations can be understood. For example, smaller MU volume and fibrosis mean less (functional) muscle fibers. Additionally, the levator muscles surround the membranous urethra and one can imagine that these muscles provide support; the thicker these muscles are and the closer these muscles are to the urethra, the more support they give to the urethra. The apical shape is divided in 4 shapes, according to Lee et al.: overlapping the membranous urethra both anteriorly and posteriorly, overlapping the membranous urethra anteriorly, overlapping membranous urethra posteriorly and no overlapping membranous urethra [25]. These shapes may influence the MUL measurement and the amount of membranous urethra that can be preserved during surgery.

On the other hand, 40 other anatomical parameters have been studied that showed no significant relation with post-prostatectomy incontinence [4].

## 4. Use of 3D Prostate MRI for Radical Prostatectomy Procedural Training and Planning

Prostate MR images can be used to guide the urologist to estimate the extent of nerve-sparing that can be achieved during radical prostatectomy, although optimal estimation is probably best performed with multivariate models [26]. MRI has been shown to change nerve-sparing plan in approximately half of the patients, compared to the plan based on clinical data only [27]. In patients planned for bilateral nerve sparing, MRI changed the strategy in 30.5%. and this was deemed accurate in 87.5–95.9% of cases [28]. Fascia preservation together with the fascia thickness seems to predict erectile function after prostatectomy [29]. Although not in all studies, several studies found lower positive surgical margins when pre-operative MRI was available [27,30].

Several parameters from standard 2D prostate MR images may be used for procedural planning. Tumor location on the MRI can predict the chance of positive surgical margins. For example, transitional zone tumors on MRI are associated with more positive surgical margins [31], as well as the involvement of the prostatic apex (31% with vs. 11% without apical involvement) [32]. Furthermore, tumor location may also influence the chance for incontinence, as an apical tumor near the membranous urethra makes it difficult to preserve the apical part of the prostatic urethra, which may decrease the chance of post-prostatectomy continence [33].

In clinical practice, extracapsular extension is assessed visually, based on bulging, irregularity, neurovascular bundle asymmetry or gross extension. Although the specificity is high, the sensitivity is low [3]. The MRI measurement of the capsular contact length, the length of the tumor contacting the capsule, may improve sensitivity. Several studies showed a relation between the capsular contact length and the extracapsular extension risk. However, highly variable cut-off values were found. For example, Mendez et al. found an optimal cut-off value of 18–24 mm [34] and Rosenkrantz et al. used 6 mm [35], both using the axial T2 sequence. Different measurement techniques have been used in the literature, e.g., MR sequence, one or more directions and linear vs. curvilinear measurement lines [36,37].

### 4.1. Three-Dimensional Models

**Figure 2:**
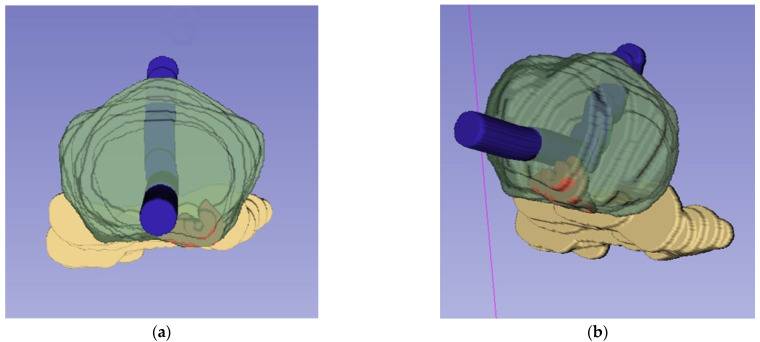
Three dimensional models used for planning and treatment of robot-assisted radical prostatectomy: a view from caudally (**a**) and a view from caudolateral (**b**); the prostate in green, the seminal vesicles in yellow and the urethra in blue. A single PI-RADS 4 focus in the peripheral zone paramedian dorsally left in the apex and midprostate (in red). The lesions were radiologically within the capsule. The neurovascular bundle was excised radically at the left side (fascia preservation score 0) and fully spared at the right side (fascia preservation score 6 out of 6). The pathological tumor stage was pT2c and the surgical margins were negative.

**Figure 3:**
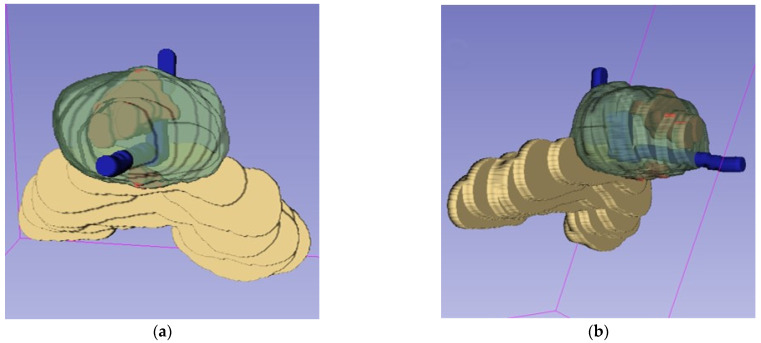
Three dimensional model used for planning and treatment of robot-assisted radical prostatectomy: a view from caudally (**a**) and a view from caudolateral (**b**); two organ-confined lesions in the midline of the prostate (red). A PI-RADS 5 focus is located in the transitional zone and anterior fibromuscular stroma anteriorly median in the apex and midprostate. A PI-RADS 4 focus is located in the peripheral zone dorsally median in the mid and base of the prostate. The neurovascular bundles were spared bilaterally, but not fully anteriorly. The fascia preservation score at the left was 5 out of 6 and right was 4 out of 6. The pathological tumor stage was pT2c and the surgical margins were negative.

A drawback of MR images is that they are a two-dimensional representation of a 3D structure. In our experience, surgeons sometimes have difficulty relating the exact tumor locations on MR images to the actual prostate in the surgical field, as humans are used to thinking and visualizing structures in three dimensions. Both 3D virtual and 3D-printed models allow urologists to understand the lesion location more accurately, but also faster and with higher level of confidence [38]. See Figure 2 and Figure 3 for examples of 3D virtual models. There is a correlation between MRI tumor and prostate volume measurements and pathology, which is an important basis for the use of 3D models [39,40,41], although MRI can underestimate tumor volume, for example, due to heterogeneous and less dense tumors [42].

#### 4.1.1. 3D Virtual Models and Augmented Reality

Ukimura et al. described the feasibility of using 3D virtual models based on both trans rectal ultrasound (TRUS) and MRI to display during RARP alongside the surgical field [43]. Samei et al. adjusted the shape of the MR images using registration with live 3D TRUS, which was also displayed alongside the image [44]. The feasibility of augmented reality (AR), superimposing the 3D images over the surgical field, was shown by several studies by Porpiglia et al. using 3D models constructed from MR images [45]. This augmented reality technique was tested by six experienced surgeons and rated the technique with 9–10 on a 10 point scale on how helpful the technique was for several important aspects of the procedure: (1) bladder neck dissection; (2) nerve-sparing technique; (3) prostate apex dissection; and (4) tailoring selective biopsies [46]. Thereafter the same study group made a technical improvement, called ‘elastic AR RAPR’, where the model was adjusted during surgery for twisting, bending, stretching and tapering [47].

#### 4.1.2. 3D-Printed Models

Additionally, the use of 3D-printed models started with several feasibility studies. Shin et al. were the first to describe the feasibility of using 3D-printed models based on MRI image delineations in planning nerve-sparing prostatectomy, later confirmed by another study [41,48]. Additionally, they found a correlation between the 3D model and histology regarding the tumor location and prostate dimensions [41]. Another group included the vessels using contrast enhanced MR images, among them the accessory pudendal arteries [49]. It has been suggested that sparing these arteries may improve functional outcome. Chandak et al. confirmed the feasibility in clinically or MRI suspected T3 tumors [50]. Porpiglia et al. showed 3D-printed model at a live surgery meeting to patients and urologist [51]. The attendees rated the models useful for surgical planning, physician education/training and patient counseling.

### 4.2. Specific Topics within 3D Model Literature

Apart from these feasibility studies, several more extensive studies have been performed on different subtopics: patient engagement, training, surgical margins, nerve-sparing surgery planning and extracapsular extension prediction. These are discussed below in subsections.

#### 4.2.1. Patient Engagement

Wake et al. studied 3D-printed, 3D virtual models and augmented reality models for patient education and compared these with the use of 2D images [52]. They found that education with 3D-printed models resulted in the best understanding of disease, cancer size, cancer location, treatment plan and the comfort level regarding the treatment plan. Understanding of size and location was better for 3D virtual models versus 2D images. In the experience of the authors, 3D models allowed better understanding of the decision to omit nerve-sparing surgery.

#### 4.2.2. Training

Witthaus et al. used 3D models to simulate nerve-sparing RARP [53]. They created 3D-printed molds from MRI scans and injected polyvinyl alcohol hydrogel into these molds. They made models of the human pelvis, bladder, prostate, urethra and neurovascular bundles. This allowed the simulation of bladder neck dissection, seminal vesicle mobilization and neurovascular bundle dissection, creating a urethrovesical anastomosis. They even measured the force to the neurovascular bundles, simulated margin status and performed a leak test of the urethrovesical anastomosis. Objective robot skills and competency were evaluated, and experts performed much better with shorter procedure time. The model was deemed suitable for training according to all the experts [53].

#### 4.2.3. Surgical Margins

In order to reduce surgical margins, it is mandatory to have good visualization of the tumor. Porpiglia et al. showed that using augmented reality, the tumor area was found at the expected level in 100% of cases [54]. Areas with extracapsular extension—but also areas of capsular abutment of the tumor—are at risk for positive surgical margins. AR-guided selective biopsies at the level of the neurovascular bundle already confirmed the extracapsular extension location in 73% [54]. Extracapsular extension and capsular abutment can together be called capsular involvement. Using augmented reality, Porpiglia et al. showed that areas of capsular involvement were correctly identified during surgery (100%). This was significantly better than using 2D MRI (47%) [47]. Positive surgical margins were lower in the 3D augmented reality group than in the 2D group (25% vs. 35%), although not statistically significant.

Checcucci et al. also studied the impact of virtual 3D models on iPad or laptop on the positive surgical margins after RARP using a matched historical cohort as control group, showing significantly lower positive surgical margins in the 3D model group (25% vs. 35%) [55]. Patients with extracapsular extension had the most advantage from using 3D models.

Bianchi et al. used augmented reality with intra-operative frozen section to guide nerve sparing RARP, compared to a control group [56]. In case of a positive frozen section, additional periprostatic tissue was resected. The positive surgical margins at the location of the index lesion were significantly lower in the augmented reality group (5% vs. 20%), although the total positive margins were not significantly lower. A recently performed randomized controlled trial showed a similar trend towards lower positive margins using 3D virtual models instead of 2D images (33% vs. 25%), although this was not statistically significant [57]. The percentage of patients with detectable post-operative PSA, however, was significantly lower in the 3D models group (9% vs. 31%) without negative consequence for functional outcomes [57].

#### 4.2.4. Nerve Sparing Planning

Estimating the extent of nerve-sparing is an essential part of preoperative surgical planning. The extent of nerve-sparing depends, apart from clinical parameters, on the size, location, capsular abutment and extracapsular extension on MRI. The interpretation by the surgeon may be challenging using 2D MR images. Our research group studied the clinical applicability of MRI based 3D prostate models in the planning of nerve-sparing robot-assisted radical prostatectomy [58]. Experienced urologists assessed 20 cases three times. In the first evaluation clinical parameters and 2D MR images were used, while in a second (3D virtual) and third (3D-printed) reading, these 3D models were added to the clinical and 2D MRI information. The planned nerve sparing was assessed using the fascia preservation score at 12 positions [59]. Clinically significant changes to the planned extent of nerve sparing were found in 25% and 26% using virtual or printed 3D models. The agreement between urologist was higher using virtual (ICC 0.52) or 3D-printed model (ICC 0.58) than 2D images (ICC 0.40), although confidence intervals overlapped [58]. Schiavina et al. studied the impact of augmented reality on nerve sparing. During pre-assessment of MRI, a plan was made on the extent of nerve sparing. After viewing the augmented reality 3D images, the plan changed in 38.5% of patients [60]. This could either be more conservative or more radical.

#### 4.2.5. Extracapsular Extension Prediction

As described above, capsular contact has been studied to predict risk of extracapsular extension. These measurements are performed in one and sometimes more directions. With 3D models, the contact surface and exact distance from the capsule can be calculated and may result in better estimating of extracapsular extension.

In our study, described at the nerve sparing section, we noted that in all seven patients with extracapsular extension the predicted location was well visualized in virtual and 3D-printed models [58]. Similar results were described in another small series [41]. We also performed a study specifically aimed to use 3D virtual models for the predictions of extracapsular extension. In this study, several clinical parameters and 3D capsular contact were used to create a model to predict extracapsular extension. The AUC of the best models for extracapsular extensions prediction was 0.802 [61]. However, this was not compared with visual assessment and capsular contact length measurements.

## 5. Discussion

In this review, we summarized the literature on the use of MRI-based (peri)prostatic anatomical measurements for improving personalized risk prediction of post-prostatectomy incontinence, most importantly the membranous urethral length. Additionally, we summarized current literature on MRI-based three-dimensional models and how it could help procedural planning of radical prostatectomy. We illustrated that much more information can be gained from prostate MRI than is commonly used in current clinical practice.

For the prediction of urinary incontinence, the MUL is the most studied parameter with the highest evidence for correlation with post-prostatectomy incontinence [4]. Therefore, the MUL is increasingly being implemented in clinical practice. However, there are several aspects of the MUL measurement to be studied and improved. Interobserver agreement studies with several studies show only ‘fair’ agreement [17,18], indicating large measurement variations. Since every millimeter of the MUL has substantial influence on the predicted incontinence risk, the observer agreement should be improved [15]. Additionally, large variations in mean/median MUL exist between published studies. Reducing measurement variations between institutions would allow the use of continence prediction nomograms and cut-off values from other institutions.

The low inter-observer agreement and large variations in mean/median MUL are likely caused by different measurement techniques. The reported measurement descriptions are very brief and poorly reproducible. Consensus among experts is needed regarding image orientation, exact landmarks used, exact line location and orientation and how the images are looked at (scrolling parasagittal and crosslinking with other, for example). Training should be available for radiologist or urologists who want to implement these measurements in their practice, and hands-on training sessions with experts in the field should be organized.

The exact manner of MUL measurement implementation can also be discussed. A certain cut-off value could be used, several risk groups (e.g., high, intermediate and low) or a nomogram that may include only the MUL or multiple parameters. In our institution, we use the CPRED nomogram developed with our own data [13]. In this nomogram, we use the MUL, the inner levator distance and predicted fascia/nerve preservation, to calculate a personalized urinary incontinence risk, which is used for shared decision-making.

Several other MRI parameters have shown significant correlations with post-prostatectomy incontinence in one or few studies [4]. These parameters should be analyzed in future studies and are not ready for large scale implementation in clinical practice.

The pre-operative planning of radical prostatectomy can be improved by MRI. The 2D MR images may already improve the planning of nerve sparing, predict risk of positive surgical margins and reduce the rate of positive surgical margins [27,28,31,32]. However, the use of 3D images is easier and faster for the urologist [38]. In the presented studies, 3D models were used for several aspect of prostatectomy: training, patient engagement, estimation of nerve saving or extracapsular extension and reducing positive surgical margins and/or postoperative PSA levels. With the use of 3D images, the inter-observer agreement for nerve sparing planning can be reduced [58]. Furthermore, 3D models can help to train urologists in performing radical prostatectomy and 3D models can be used to help patients to understand their procedure. Importantly, by using 3D models instead of 2D MR images, positive surgical margins and post-surgical PSA levels can be further reduced, which will reduce the risk of recurrence [55,57]. However, the evidence is still limited for all studied aspects and more research is needed. Especially the promising results on reducing surgical margins and post-surgical PSA warrant a well-designed randomized clinical trial of sufficient power. Preferably, these trials should study the effect of 3D models on both oncological and functional outcome.

## 6. Future Directions

The use of artificial intelligence for automated MUL measurements may offer a solution for the variable inter-observer agreement, which is important for clinical implementation. A robust AI algorithm that can handle various MR images from different field strength machines, different vendors and different T2 sequences may be a solution. A urinary incontinence risk nomogram developed on the same dataset as the AI algorithm can be used.

The use of artificial intelligence may also facilitate clinical implementation of 3D models for pre-operative planning. Manual delineations of the prostate, the tumor and other structures, such as the urethra and seminal vesicles, are very labor-intensive. Automatic delineations by artificial intelligence can already be produced by commercially available software packages. Delineations can be checked and approved by the radiologist.

For now, model orientation during augmented reality assisted prostatectomy is performed manually, which is labor-intensive and probably suboptimal for the orientation. Artificial intelligence may be able to automatically correct orientation during surgery in the future, increasing the speed and accuracy of the orientation, reducing the labor and therefore costs.

## 7. Conclusions

The membranous urethral length measured on the preoperative prostate MRI can predict post-prostatectomy incontinence risk in order to facilitate shared decision-making based on individualized risk and thereby improving the patient selection for radical prostatectomy. MRI-based 3D virtual or 3D-printed models show promising results for improving several aspects of procedural radical prostatectomy planning and may lead to improved patient outcomes, and therefore, more research on this topic is warranted.

## Figures and Tables

**Table 1:** Summary of parameters with significant correlation with urinary incontinence in one or few studies.

Prostate Related	Urethra Related	Musculoskeletal
apical protrusion (overlapping membranous urethra)	thicker urethra wall thickness	larger inner or outer levator distance
greater prostate length/depth	severe urethral fibrosis	thinner levator ani muscle thickness
>5 mm intravesical prostatic protrusion	smaller membranous urethra volume	lower levator ani muscle perfusion ratio
	larger angle between the MUL and the prostate axis	shorter pelvic diaphragm length
	shorter minimal residual MUL	small dorsal vascular complex

## Data Availability

Not applicable.

## Appendix A

**Table A1:** Search used in our recent systematic review [4] and updated to 5 August 2022: (Date of inception after each database).

Database Searched	Records	Records after Duplicates Removed
Embase.com (1971-)	1350	1311
Medline ALL Ovid (1946-)	431	78
Web of Science Core Collection (1975-)	395	60
Cochrane CENTRAL register of trials (1992-)	11	23
Google scholar	100	45
Total	2307	1505

### Appendix A.1. Embase.com (1971-)

(‘prostate tumor’/exp/dm_su OR ‘prostate tumor’/exp/dm_rt OR (‘prostate tumor’/exp AND (‘focal therapy’/de OR ‘cryoablation’/de OR ‘cancer surgery’/de OR ‘active surveillance’/de OR ‘watchful waiting’/de OR ‘cryotherapy’/exp OR radiotherapy/exp)) OR ‘prostatectomy’/exp OR (((prostat*) NEAR/6 (cancer* OR carcinom* OR adenocarcinom* OR neoplas* OR tumo* OR lesion* OR oncolog*) NEAR/10 (surg* OR operat* OR postsurg* OR postoperat* OR radiotherap* OR irradiation* OR radiolog* OR focal-therap* OR cryotherap* OR cryosurg* OR cryoablat* OR cryo-ablat* OR Brachytherap* OR active-surveillan* OR watchful-wait*)) OR prostatectom* OR (prostat* NEAR/3 (resection OR adenectom*))):ab,ti) AND (‘nuclear magnetic resonance imaging’/exp OR ‘nuclear magnetic resonance’/exp OR (mri OR mpmri OR (MR NEAR/3 imag*) OR (magnetic* NEAR/3 resonan*)):ab,ti) AND (‘urinary continence’/de OR ‘urine incontinence’/exp OR incontinence/de OR continence/de OR ‘functional outcome’/exp OR ‘urine retention’/de OR ‘International Prostate Symptom Score’/de OR ‘micturition’/de OR ‘micturition disorder’/de OR (continen* OR incontinen* OR retention OR (function* NEAR/3 outcome*) OR micturition OR urination):ab,ti) NOT ([conference abstract]/lim AND [1800-2017]/py) AND [English]/lim NOT ([animals]/lim NOT [humans]/lim)

### Appendix A.2. Medline ALL Ovid (1946-)

(exp Prostatic Neoplasms/su OR exp Prostatic Neoplasms/rt OR (exp Prostatic Neoplasms/AND (Cryosurgery/OR Watchful Waiting/OR Cryotherapy/OR Radiotherapy/)) OR Prostatectomy/OR (((prostat*) ADJ6 (cancer* OR carcinom* OR adenocarcinom* OR neoplas* OR tumo* OR lesion* OR oncolog*) ADJ10 (surg* OR operat* OR postsurg* OR postoperat* OR radiotherap* OR irradiation* OR radiolog* OR focal-therap* OR cryotherap* OR cryosurg* OR cryoablat* OR cryo-ablat* OR Brachytherap* OR active-surveillan* OR watchful-wait*)) OR prostatectom* OR (prostat* ADJ3 (resection OR adenectom*))).ab,ti.) AND (exp Magnetic Resonance Imaging/OR exp Magnetic Resonance Spectroscopy/OR (mri OR mpmri OR (MR ADJ3 imag*) OR (magnetic* ADJ3 resonan*)).ab,ti.) AND (exp Urinary Incontinence/OR Urinary Retention/OR Urination/ORUrination Disorders/OR (continen* OR incontinen* OR retention OR (function* ADJ3 outcome*) OR micturition OR urination).ab,ti.) AND english.la. NOT (exp animals/NOT humans/)

### Appendix A.3. Web of Science Core Collection (1975-)

TS=(((((prostat*) NEAR/5 (cancer* OR carcinom* OR adenocarcinom* OR neoplas* OR tumo* OR lesion* OR oncolog*) NEAR/10 (surg* OR operat* OR postsurg* OR postoperat* OR radiotherap* OR irradiation* OR radiolog* OR focal-therap* OR cryotherap* OR cryosurg* OR cryoablat* OR cryo-ablat* OR Brachytherap* OR active-surveillan* OR watchful-wait*)) OR prostatectom* OR (prostat* NEAR/2 (resection OR adenectom*)))) AND ((mri OR mpmri OR (MR NEAR/2 imag*) OR (magnetic* NEAR/2 resonan*))) AND ((continen* OR incontinen* OR retention OR (function* NEAR/2 outcome*) OR micturition OR urination))) AND DT = (article) AND LA = (english)

### Appendix A.4. Cochrane CENTRAL Register of Trials (1992-)

((((prostat*) NEAR/6 (cancer* OR carcinom* OR adenocarcinom* OR neoplas* OR tumo* OR lesion* OR oncolog*) NEAR/10 (surg* OR operat* OR postsurg* OR postoperat* OR radiotherap* OR irradiation* OR radiolog* OR focal-therap* OR cryotherap* OR cryosurg* OR cryoablat* OR cryo-ablat* OR Brachytherap* OR active-surveillan* OR watchful-wait*)) OR prostatectom* OR (prostat* NEAR/3 (resection OR adenectom*))):ab,ti) AND ((mri OR mpmri OR (MR NEAR/3 imag*) OR (magnetic* NEAR/3 resonan*)):ab,ti) AND ((continen* OR incontinen* OR retention OR (function* NEAR/3 outcome*) OR micturition OR urination):ab,ti)

### Appendix A.5. Google Scholar

“prostate|prostatic cancer|carcinoma|adenocarcinoma|neoplasms|tumors” surgery|operative|radiotherapy|cryotherapy|cryoablation|prostatectomy mri|”magnetic resonance” continence|incontinence|continent|incontinent
